# Supplementary material for: Splice-Junction-Based Mapping of Alternative Isoforms in the Human Proteome
Source: Cell Rep. Author manuscript; Available in PMC 2020 Jan 15. (PMC6961840; doi:10.1016/j.celrep.2019.11.026)
Supplement: 2 [file NIHMS1546469-supplement-2.zip › DF1/PXD006675/RightAtrium/RightAtrium_9_TTN_APHVEFLRPLTDLQVR.pdf]

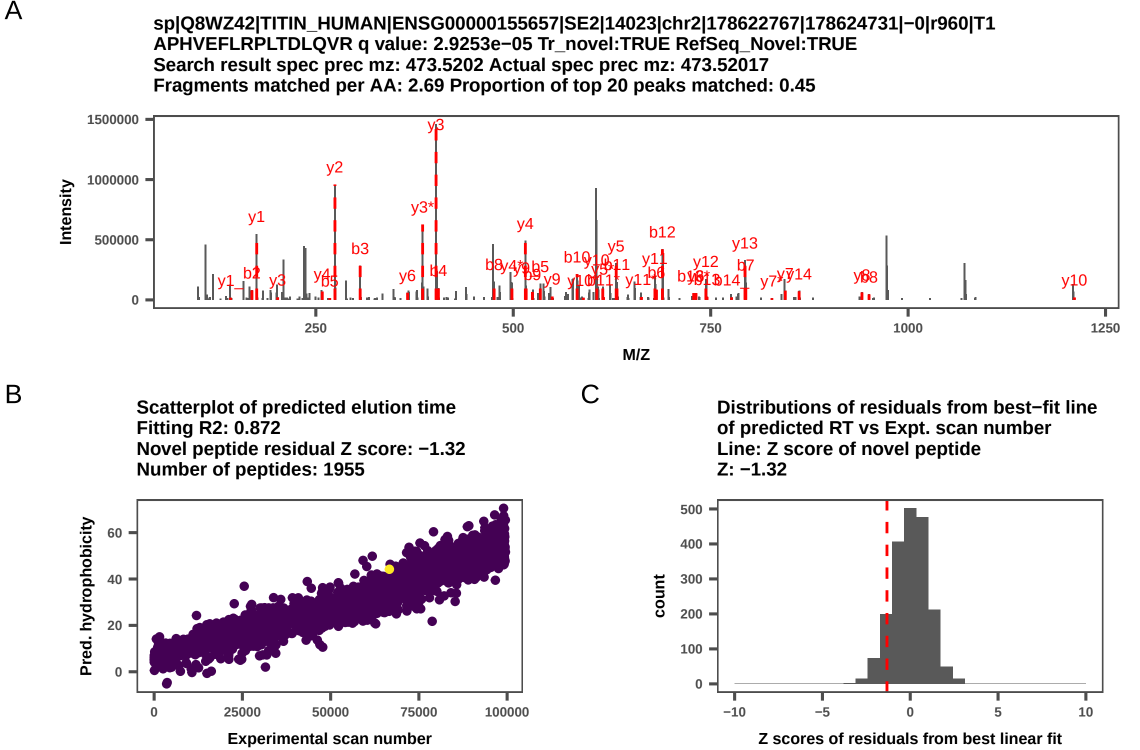

A
sp|Q8WZ42|TITIN_HUMAN|ENSG00000155657|SE2|14023|chr2|178622767|178624731|−0|r960|T1
APHVEFLRPLTDLQVR q value: 2.9253e−05 Tr_novel:TRUE RefSeq_Novel:TRUE
Search result spec prec mz: 473.5202 Actual spec prec mz: 473.52017
Fragments matched per AA: 2.69 Proportion of top 20 peaks matched: 0.45
Intensity
1500000
1000000
500000
0
M/Z
250
500
750
1000
1250
y3
y2
y3*
y1
y4
b12
b3
y13
b4
y6
b7
y10
B
Scatterplot of predicted elution time
Fitting R2: 0.872
Novel peptide residual Z score: −1.32
Number of peptides: 1955
Pred. hydrophobicity
60
40
20
0
Experimental scan number
0
25000
50000
75000
100000
C
Distributions of residuals from best−fit line
of predicted RT vs Expt. scan number
Line: Z score of novel peptide
Z: −1.32
count
500
400
300
200
100
0
Z scores of residuals from best linear fit
−10
−5
0
5
10
